# Effects of exogenous calcium on the drought response of the tea plant (*Camellia sinensis* (L.) Kuntze)

**DOI:** 10.7717/peerj.13997

**Published:** 2022-08-29

**Authors:** Lyudmila S. Malyukova, Natalia G. Koninskaya, Yuriy L. Orlov, Lidiia S. Samarina

**Affiliations:** 1 Federal Research Centre the Subtropical Scientific Centre of the Russian Academy of Sciences, Sochi, Russia; 2 Agrarian and Technological Institute, Peoples’ Friendship University of Russia, Moscow, Russia; 3 Digital Health Institute, I.M. Sechenov First Moscow State Medical University of the Ministry of Health of the Russian Federation (Sechenov University), Moscow, Russia; 4 Center of Genetics and Life Sciences, Sirius University of Science and Technology, Sochi, Russia

**Keywords:** Tea plant, Plant physiology, Drought stress, Exogenous calcium, Plant transcription factors

## Abstract

**Background:**

Drought is one of the major factors reducing the yield of many crops worldwide, including the tea crop (*Camellia sinensis* (L.) Kuntze). Calcium participates in most of cellular signaling processes, and its important role in stress detection and triggering a response has been shown in many crops. The aim of this study was to evaluate possible effects of calcium on the tea plant response to drought.

**Methods:**

Experiments were conducted using 3-year-old potted tea plants of the best local cultivar Kolkhida. Application of ammonium nitrate (control treatment) or calcium nitrate (Ca treatment) to the soil was performed before drought induction. Next, a 7-day drought was induced in both groups of plants. The following physiological parameters were measured: relative electrical conductivity, pH of cell sap, and concentrations of cations, sugars, and amino acids. In addition, relative expression levels of 40 stress-related and crop quality–related genes were analyzed.

**Results:**

Under drought stress, leaf electrolyte leakage differed significantly, indicating greater damage to cell membranes in control plants than in Ca-treated plants. Calcium application resulted in greater pH of cell sap; higher accumulation of tyrosine, methionine, and valine; and a greater Mg^2+^ content as compared to control plants. Drought stress downregulated most of the quality-related genes in both groups of tea plants. By contrast, significant upregulation of some genes was observed, namely *CRK45*, *NAC26*, *TPS11*, *LOX1*, *LOX6*, *Hydrolase22*, *DREB26*, *SWEET2*, *GS*, *ADC*, *DHN2*, *GOLS1*, *GOLS3*, and *RHL41*. Among them, three genes (*LOX1*, *RHL41*, and *GOLS1*) showed 2–3 times greater expression in Ca-treated plants than in control plants. Based on these results, it can be speculated that calcium affects galactinol biosynthesis and participates in the regulation of stomatal aperture not only through activation of abscisic-acid signaling but also through jasmonic-acid pathway activation. These findings clarify calcium-mediated mechanisms of drought defense in tree crops. Thus, calcium improves the drought response in the tea tree.

## Introduction

High yields under unfavorable environmental conditions are important for sustainable crop production. Drought is a major environmental constraint reducing the yield of many economically important crops, and climate aridization has been increasing worldwide. One negative effect of drought stress is oxidative damage leading to disturbances of physiological and biochemical processes causing significant losses in tea quality and yields (Upadhyaya & Panda, 2004; Marcińska et al., 2013; Maritim et al., 2015). During earlier research, many genetic and physiological mechanisms of drought tolerance have been clarified in various crops including the tea crop (Bhagat, Baruah & Cacigue, 2010; Damayanthi, Mohotti1 & Nissanka, 2010; Man et al., 2011; Baruah & Bhagat, 2012; Maritim et al., 2015; Zhu, 2016; Fleta-Soriano & Munné-Bosch, 2016; Malyukova et al., 2020, 2021; Samarina et al., 2020). It has been shown that plants reorganize their osmoregulatory and antioxidant systems and secondary-metabolite production in response to drought stress (Fleta-Soriano & Munné-Bosch, 2016; Zhu, 2016). Many transcription factors and metabolite-related genes are involved in the drought response in the tea plant. For example, key cold response regulators *ICE*, *CBF*, and *DHN* participate in the drought response too (Liu et al., 2016a; Yin et al., 2016; Ban et al., 2017; Hu et al., 2020). Transcriptomic data on the tea plant have revealed 12 transcription factor families (AP2/EREBP, bHLH, bZIP, HD-ZIP, HSF, MYB, NAC, WRKY, zinc-finger protein transcription factors, SCL, ARR, and SPL) performing crucial functions in tea drought responses *via* both abscisic acid (ABA)-dependent and ABA-independent pathways (Liu et al., 2016a; Yue et al., 2015; Wang et al., 2016a; Cui et al., 2018; Chen et al., 2018; Ma et al., 2019; Samarina et al., 2020).

Although many mechanisms of tea drought responses have been revealed, the topic of exogenous regulation of drought tolerance by chemical and biological compounds is still not elucidated sufficiently. Some studies indicate enhancement of drought tolerance by hormone treatments (Man et al., 2011; Njoloma, 2012; Upadhyaya & Panda, 2004). On the other hand, external application of mineral nutrients to increase drought tolerance still has not been studied well. Among a wide range of biogenic macro- and microelements, calcium is of particular interest because it participates in signal transduction under unfavorable environmental conditions (Zhang et al., 2015; Edel et al., 2017; Singh, Parihar & Prasad, 2018; Thor, 2019; Hosseini et al., 2019; Ramírez-Builes et al., 2020). Calcium takes part in most of cellular signaling processes, and its important role in early stress detection and triggering a response has been demonstrated in many crops (Kim, 2009; Upadhyaya et al., 2012; Singh, Parihar & Prasad, 2018; Thor, 2019; Hosseini et al., 2019). Calcium interacts strongly with reactive oxygen species and participates in H_2_O_2_ sensing and in the induction of antioxidant defense in plants (Rentel & Knight, 2004; Evans et al., 2005; Noctor, 2006). It is believed that calcium influx and cytoplasmic calcium ([Ca^2+^]_cyt_) are important for ABA transduction in guard cells, whereas ABA can regulate stomatal aperture in guard cells. In *Arabidopsis thaliana*, amplitudes of extracellular Ca^2+^ concentration oscillation and of cytosolic Ca^2+^ concentration oscillation are controlled by soil Ca^2+^ levels and transpiration rates (Hetherington & Brownlee, 2004; Kim, 2009).

Although external calcium application has been useful at increasing abiotic-stress tolerance in several crops, it is not usually considered a tool for improving drought tolerance of tea plantations because tea is an acidophilic crop. Nevertheless, a deficiency of soil calcium was revealed during our long-term observations in tea plantations on the Black Sea Coast of the Caucasus. Soil acidification usually develops during long-term cultivation of tea on acidic soils, thereby significantly diminishing the amounts of available forms of calcium (Malyukova et al., 2021). Thus, the aim of the present study was to assess a possible effect of external calcium application on the drought response of the tea plant in terms of physiological parameters and gene expression levels.

## Materials and Methods

### Plant materials, growth conditions, and stress induction

Three-year-old vegetatively propagated potted plants of the best local tea cultivar Kolkhida were used in this study (Fig. S1). Plants were 50 cm tall and grown in 2-liter polyethylene pots filled with brown forest acidic soil. Before the drought treatment, the experimental plants were precultivated under controlled conditions for 15 days at 20 °C ± 2 °C, humidity of 60% ± 5%, light intensity of 5,000 lux, on a 16/8 h light/dark cycle. At the beginning of this period, one of two fertilizer treatments was started.

– Control treatment: four-time application of an ammonium nitrate solution, 50 ml (150 mg/l, which is equivalent to 100 mg of nitrogen per plant) per pot of soil, during a month: days 0, 10, 20, and 30. Soil pH was 3.9.
– Cа treatment: four-time application of a calcium nitrate solution, 50 ml (400 mg/l, which is equivalent to 100 mg of nitrogen and 150 mg of calcium per plant) per pot of soil during the month: days 0, 10, 20, and 30. Soil pH was 4.3.

On day 30, leaves were sampled for physiological, biochemical, and gene expression analyses and were designated as “no drought” treatment groups. After that, these plants were subjected to soil drought *via* a gradual decrease in watering: during 7 days, soil humidity was reduced from 70% ± 2% to 16% ± 2%, until cell sap concentration reached a critical level of 10–12%. Next, leaves were sampled for physiological, biochemical, and gene expression analyses and were designated as “drought” treatment groups.

For each analysis, 2^nd^, 3^rd^, and 4^th^ mature leaves from the top of a plant were sampled in the morning between 8 and 9 am. All experiments were performed on three biological replicates (three plants); all experiments were conducted twice in the 2019–2020 period.

### Assays of physiological and biochemical parameters

The leaf water content was calculated as $C = \left( {\displaystyle{{\left( {FW - DW} \right)} \over {FW}}} \right)*100\%$, where FW is fresh leaf mass, and DW is dried leaf mass (leaves were dried at 105 °C in an oven for 5 h) (Yamasaki & Dillenburg, 1999).

Electrolyte leakage was determined using a ST300C portable conductivity meter (Ohaus, USA) *via* the following formula:

$EL = \displaystyle{{\left( {\displaystyle{{L1} \over {L0}}} \right)} \over {\left( {\displaystyle{{L2} \over {L0}}} \right)}}*100,$

where L0 and L1 are electrical conductivity immediately and 2 h after leaf immersion in deionized water, respectively, and L2 is conductivity after boiling for 120 min at 100 °C with subsequent cooling to room temperature (Bajji, Kinet & Lutts, 2001).

pH of cell sap was determined by means of a Testo 205 pH-meter (Moscow, Russia) with a hydrogen electrode. For this measurement, 1,000 mg of fresh leaf tissue was homogenized in 20 ml of distilled water (Malyukova et al., 2020).

Amino acids (mg g^−1^ dried leaf mass), sugars (mg g^−1^ dried leaf mass) and cations (µg g^−1^ dried leaf mass) and organic acids (mg g^−1^ dried leaf mass) were assayed by capillary electrophoresis on a Kapel-105M analyzer (Russia) (Brykalov et al., 2019).

### Analyses of gene expression profiles by quantitative reverse-transcription PCR (qRT-PCR)

Total RNA was extracted from the third mature leaf in three biological replicates by the guanidine method according to the manufacturer’s protocol (Biolabmix, Novosibirsk, Russia; https://biolabmix.ru/). The concentration and quality of RNA were determined on a Bio-drop µLite spectrophotometer (Biochrom, Cambridge, UK) and RNA integrity was assessed by electrophoresis in a 1% agarose gel. Then, 1,000 ng of RNA was treated with 1 µl of DNaseI Buffer and 1 µl DNaseI (Biolabmix, Novosibirsk, Russia; https://biolabmix.ru/) for 30 min at 42 °C with subsequent DNase inhibition by heating. After that, 1,000 ng of RNA was employed to prepare 20 µl of cDNA using the M-MuLV–RH-kit (Biolabmix, Novosibirsk, Russia; https://biolabmix.ru/) with subsequent quality evaluation by gel electrophoresis and qRT-PCR on LightCycler96 (Roche Life Sciences, Penzberg, Germany; https://lifescience.roche.com/global_en.html). After cDNA preparation, all samples were diluted to the same concentration of 700 ng µl^−1^ according to standardization by means of the expression of a reference gene, actin (NCBI Gene ID: 114316878). To measure gene expression, qPCR was carried out in a 15 µl reaction mixture consisting of 7.5 µl of 2× SybrBlue qRT-PCR buffer with hot-start polymerase (Biolabmix, Novosibirsk, Russia; https://biolabmix.ru/), 0.2 µl of each primer (forward and reverse), 1 µl of cDNA, and the rest of the volume was PCR grade water. A two-step amplification program was as follows: preheating for 5 min at 95 °C, 40 cycles of amplification (10 s at 95 °C and 30 s at 56–62 °C), final extension for 5 min at 72 °C, and melting for 3 min at 95 °C. In total, more than 40 genes were analyzed in this study (Table S1).

The relative gene expression level was calculated by the 2^−∆∆Cq^ method of Livak & Schmittgen (2001), where

$\Delta\Delta{\rm Cq} = ({\rm Cq}_{gene\;of\; interest} - {\rm Cq}_{internal\; control})_{treatment} - ({\rm Cq}_{gene \;of\; interest} - {\rm Cq}_{internal\; control})_{control.}$

### Data analysis and visualization

The experimental design was completely randomized. One-way ANOVA and Student’s *t* test were performed to find significant differences in effects among the treatments. The significance of the differences was evaluated by the Fisher test, LSD_05_, and standard deviations from the mean. In addition, principal component analysis (PCA) and hierarchical clustering were conducted to examine the relations and visualize genetic and biochemical results. Dissimilarities were calculated using the DICE coefficient, with agglomeration by Ward’s method. Two separate matrices of biochemical and the genetic data were subjected to PCA. Before this procedure, the data were normalized: all data were converted to the ratio of a drought treatment group to a no-drought treatment group. After that, the normalized matrices were analyzed separately by Pearson (n)-type PCA, and two plots (biochemical and genetic) were superimposed. Statistical analyses of the data were carried out in the XLSTAT software (free trial version) (https://www.xlstat.com/).

## Results

### The effect of Ca application on physiological and biochemical parameters of the tea plant under drought

**Figure 1:**
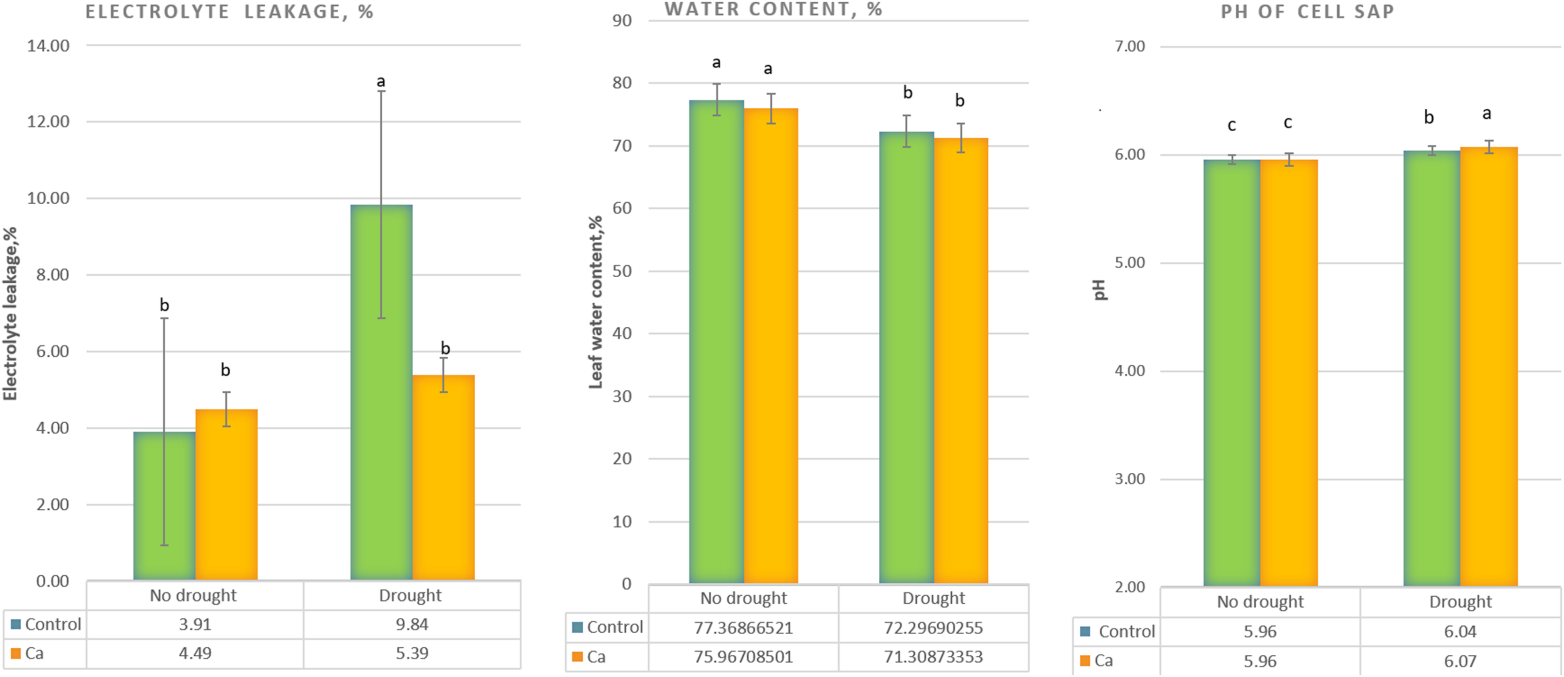
Physiological parameters of the tea tree under drought. Relative electrical conductivity (A), leaf water content (B), and pH of cell sap (C). Different lowercase letters indicate significant differences at *P* < 0.05.

Under drought stress, three-time elevation of electrolyte leakage was noted in control plants but not in Ca-treated plants (Fig. 1A). A significant decrease in the water content (from 76–77% to 71–72%) was detected in both control and Ca-treated plants (Fig. 1B). Furthermore, pH of cell sap increased significantly under drought stress in both groups of plants, and Ca-treated plants experienced higher elevation of pH as compared to control plants (Fig. 1C).

**Figure 2:**
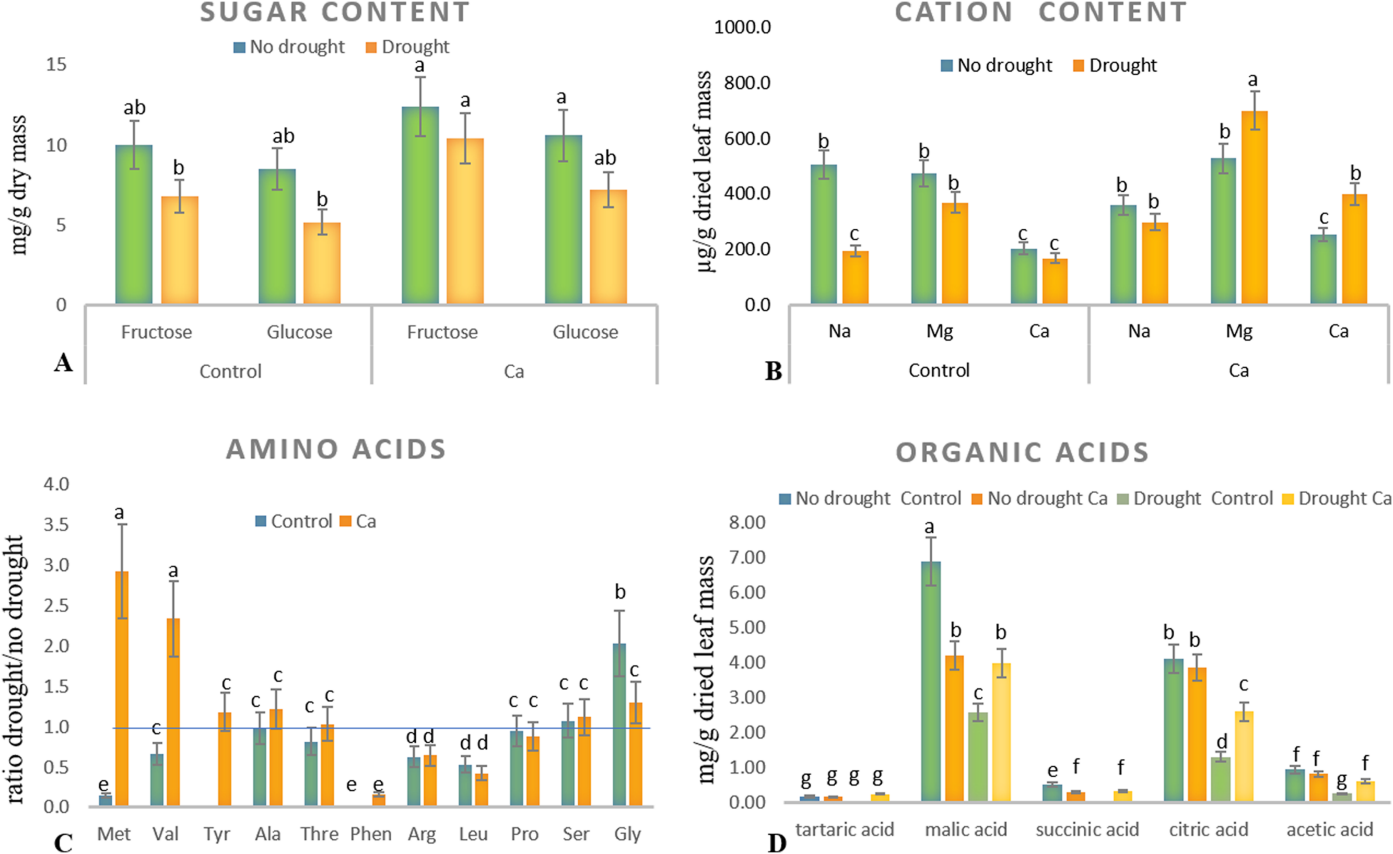
Biochemical parameters in the tea tree under drought. Accumulation of biochemical compounds in mature tea leaves under drought stress: sugars (A), cations (B), amino acids (C), and organic acids (D). Different lowercase letters indicate significant differences at *P* < 0.05.

As for biochemical parameters, Ca-treated plants manifested significantly higher glucose (23 mg g^−1^) and fructose (17.6 mg g^−1^) accumulation as compared to control plants (18.6 and 11.9 mg g^−1^, respectively) under drought (Fig. 2A). Additionally, in both groups of plants, cation contents changed significantly in tea leaves under drought (Fig. 2B). That is, control plants showed a significant decrease in the Na^+^ content from 506 to 193 µg g^−1^ dry leaf mass, which was not observed in Ca-treated plants, but significant elevation of the Ca^2+^ content from 253 to 398 µg g^−1^ dry leaf mass and increased Mg^2+^ accumulation were registered in Ca-treated plants under drought.

Drought stress diminished the content of several amino acids, namely Met, Val, Phen, Arg, and Leu, in mature tea leaves. In Ca-treated tea plants, Met, Val, and Tyr accumulated more strongly under drought stress (Fig. 2C). Among these compounds, the highest accumulation was detected for two amino acids (Met (3-fold) and Val (2-fold)) in comparison with no-drought conditions. The levels of Pro, Ser, Thr, and Ala were not influenced either by Ca application or by drought induction.

Different levels of organic acids were present before drought induction: control plants had 1.5–2.0-fold higher levels of malic, citric, succinic, and acetic acids than the Ca-treated plants did (Fig. 2D). Under drought stress, contents of malic, succinic, citric, and acetic acids declined 2–3-fold in control plants. By contrast, only the citric acid content decreased in Ca-treated plants. To summarize, drought treatment significantly affected physiological integrity of tea plants, but Ca-treated and control plants showed different responses to drought, indicating better physiological status under stress in Ca-treated plants.

### The impact of Ca application on gene expression profiles of the tea plant under drought and relations with biochemical data

**Figure 3:**
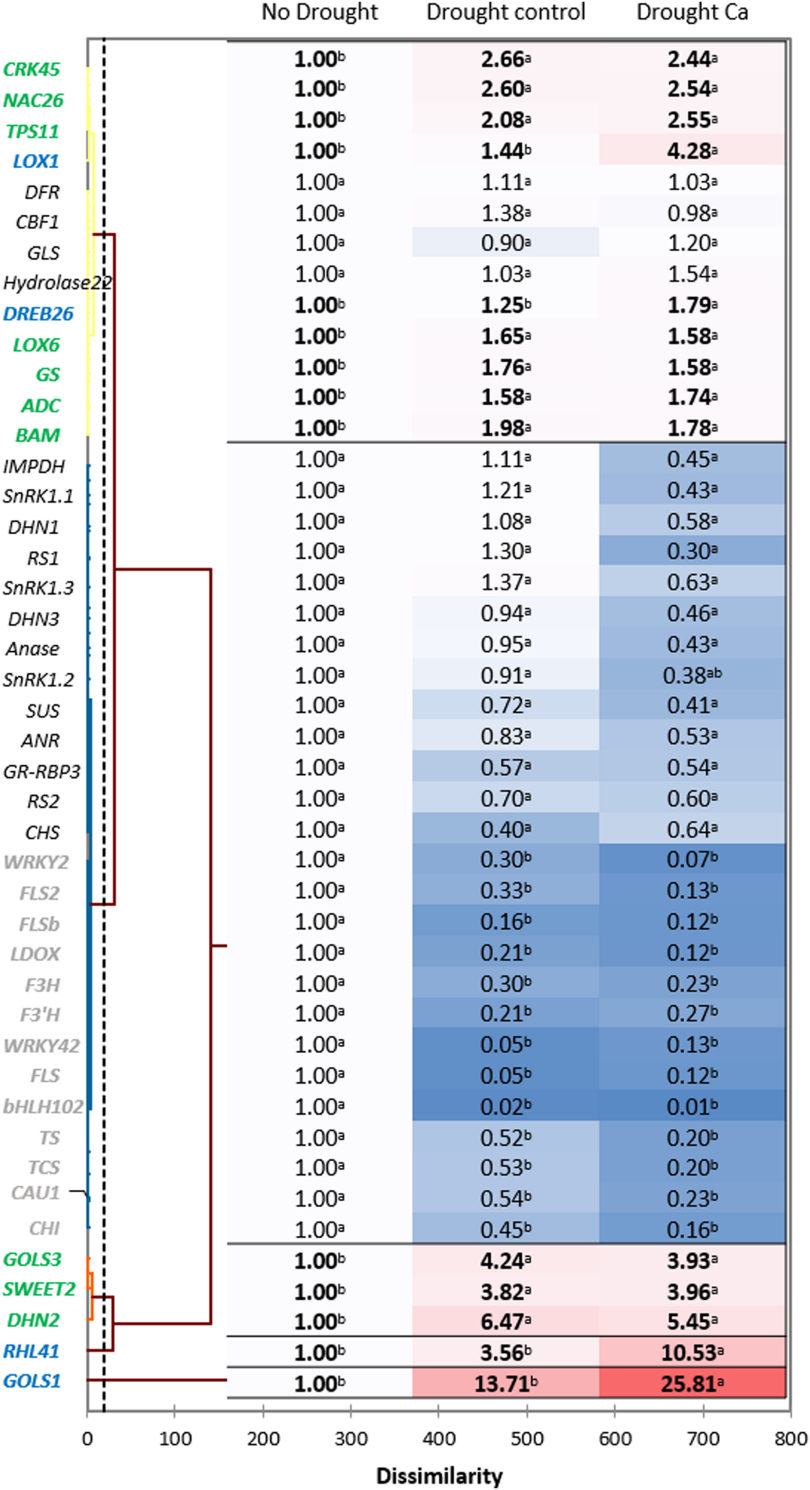
Gene expression in the tea tree under drought. A heat map of relative gene expression and hierarchical clustering of genes in the tea plant under drought stress. Genes highlighted in green: upregulated under drought in both groups of plants *vs*. no drought; blue: genes with greater upregulation in Ca-treated plants; orange: genes with greater upregulation under drought in control plants; gray: genes with decreased expression in both groups of plants *vs*. no drought. Different lowercase letters indicate significant differences at *P* < 0.05.

Hierarchical clustering based on the gene expression profiles gave five distant clusters (Fig. 3). The upper cluster contained 13 genes including nine genes upregulated by drought (*CRK45*, *NAC26*, *TPS11*, *LOX1*, *DREB26*, *LOX6*, *GS*, *ADC*, and *BAM*).

The second cluster was the biggest one and comprised 26 genes. Among them, 13 genes (*WRKY2*, *FLS2*, *FLSb*, *LDOX*, *F3’H*, *F3H*, *WRKY42*, *FLS*, *bHLH102*, *TCS*, *TS*, *CAU1*, and *CHI*) were downregulated by drought stress, and the other 13 genes (*SnRK1.1*, *SnRK1.2*, *SnRK1.3*, *IMPDH*, *DHN1*, *RS1*, *Anase*, *DHN3*, *ANR*, *SUS*, *RS2*, *GR-RBP3*, and *CHS*) were not affected by drought.

The third cluster consisted of three genes with increased expression under drought: *GOLS3*, *SWEET2*, and *DHN2*. The greatest fold change was registered for two genes—*RHL41* and *GOLS1*—which were outliers.

Finally, among the drought-induced genes, four genes (*RHL41*, *GOLS1*, *LOX1*, and *DREB26)* were more strongly expressed in Ca-treated plants than in control plants.

**Figure 4:**
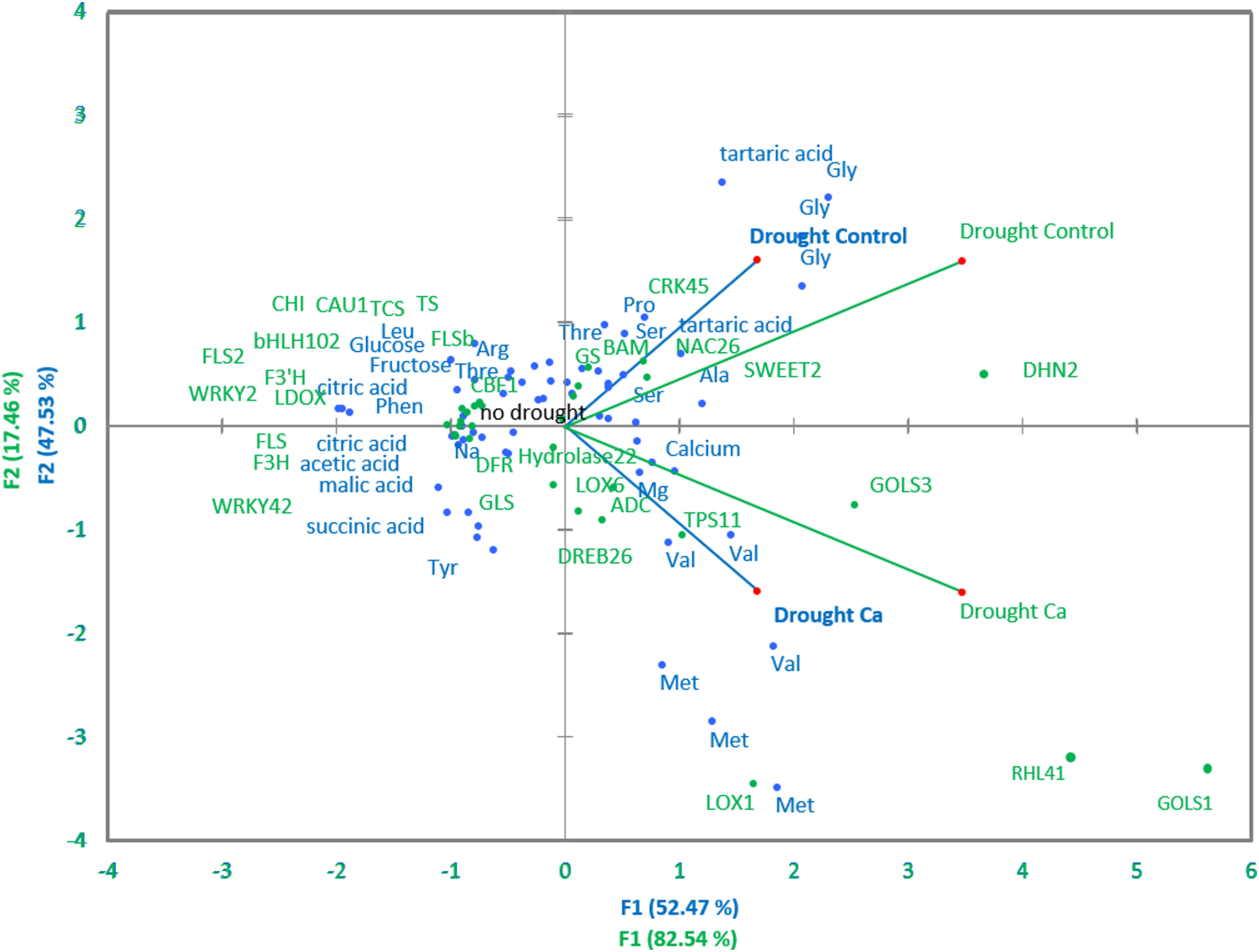
PCA of biochemical and genetic parameters. A PCA biplot representing superimposed data from biochemical and genetic principal component analyses in Ca-treated (Ca) and Ca-untreated (Control) tea plants under drought. Green: genetic data, blue: biochemical data.

Pairwise comparisons of the four treatment groups by PCA uncovered high Pearson’s correlation between two principal components and factors at a significance level of α = 0.05. Several genes and biochemical parameters clearly separated two groups (Drought-Control and Drought-Ca) with high positive loadings (Fig. 4). The largest square cosines (0.78) were observed in principal component 1 (PC1). Examination of the biplot (superimposed PCA plots) revealed that the Drought-Control and Drought-Ca vectors are positioned on the positive and negative sides of PC1, respectively, with high loadings. Several data points related to each vector were found to be grouped closely with them. For example, the highest positive loading belongs to genes *RHL41*, *GOLS1* and *LOX1*, and amino acids Met and Val are distributed with high loading along the Drought-Ca vector. In addition, many genes and biochemical parameters that were more pronounced in the control (no drought) treatment group are densely clustered and located on the PC2 side, orthogonally to the drought-related vectors. Nonetheless, most of PC2 scores are low, meaning weak correlations between the data points.

## Discussion

### The effect of Ca application on physiological and biochemical parameters of the tea plant under drought

In this study, we assessed the effect of calcium application to soil on the tea plant drought response. Under drought stress, typical physiological changes were observed in plants such as alterations in the leaf water content, electrolyte leakage, and pH of cell sap, which are prominent phenotypical indicators of the drought response in plants. Nonetheless, Ca-treated plants manifested less electrolyte leakage and elevated pH, pointing to less damage to cell membranes under drought stress. Cell sap pH is the one of the first signals leading to ABA synthesis and stomata closure; therefore, it is a sensitive physiological indicator of the drought response (Song et al., 2008), consistently with our results. The higher concentration of sugars observed in Ca-treated tea plants before stress induction is probably due to predominance of the sucrose biosynthetic pathway over the starch biosynthetic pathway; this arrangement can ensure higher viscosity of the cytoplasm to prevent water evaporation under drought, in agreement with previous data (Kaelke & Dawson, 2005; Kasuga, Arakawa & Fujikawa, 2007; Regier et al., 2010).

Among the other physiological parameters, the cation content is an important diagnostic parameter for site-specific and efficient nutrient management (Hauer-Jákli & Tränkner, 2019). Our results uncovered drought-induced elevation of the Mg^2+^ content in Ca-treated plants; this alteration can provide better antioxidant defense under drought stress, as reported by some researchers (Hauer-Jákli & Tränkner, 2019). Magnesium, the most abundant free divalent cation in the cell, participates in carbon fixation and photosynthesis. It acts as an activator or cofactor of enzymes in carbohydrate metabolism and plays an important part in photo-oxidative defense (Guo et al., 2015; Hauer-Jákli & Tränkner, 2019; Grzebisz, 2013; Chen et al., 2018). On the other hand, some studies indicate antagonistic relations between Mg^2+^ and Ca^2+^ in plants (Gransee & Führs, 2013), a synergistic effect of Ca^2+^ and K^+^ uptake, and no influence on Mg^+2^ uptake in the coffee plant (Ramírez-Builes et al., 2020). Nonetheless, these studies mostly describe competitive absorption of Mg^2+^ and Ca^2+^ from the soil fertilized with these nutrients. In our work, the Mg^2+^ accumulation can be explained by relocation of the magnesium ions transported from other organs into leaves; this process was positively affected by Ca^2+^ application.

Accumulation of amino acids is a well-known mechanism of osmotic adjustment, of detoxification of reactive oxygen species, and of intracellular pH regulation under various osmotic stresses (Silvente, Sobolev & Lara, 2012). Among different amino acids, Met and Val accumulated to a greater extent under drought stress in our Ca-treated tea plants than in control tea plants. Both are protein-bound amino acids and play an important role in plant metabolism (Jander & Joshi, 2010; Binder, 2010; Hildebrandt, 2018). Aspartate-derived amino acid Met is tightly connected with the metabolism of branched-chain amino acids Val, Leu, and Ile, which activate jasmonic acid (JA) signaling; the latter is crucial for plants’ resistance to biotic and abiotic stressors (Jander & Joshi, 2010; Binder, 2010). Thus, higher accumulation of Met and Val in the tea plant may be one more piece of evidence for activation of JA signaling by Ca^2+^ under drought. Of note, under drought stress, Gly accumulation was higher in control plants than in Ca-treated plants. Gly is a major component of glycine-rich proteins and is involved in RNA post-transcriptional processing, including splicing and polyadenylation, which are believed to perform a crucial function in plants’ responses to abiotic stressors (Khan et al., 2017; Czolpinska & Rurek, 2018). Further studies are necessary to assess the role of Gly and of the Gly metabolic pathway in the tea plant under drought conditions.

Organic-acid metabolism not only equilibrates redox potential in plant cells but also transfers redox equivalents between cell compartments thereby supporting various metabolic processes (Igamberdiev & Eprintsev, 2016; Igamberdiev & Bykova, 2018). Here, before drought induction, several organic acids (malic, citric, succinic, and acetic) steadily accumulated in control plants in comparison with Ca-treated plants (Fig. 2D). By contrast, under subsequent drought stress, the contents of malic, succinic, citric, and acetic acids diminished in control plants but not in Ca-treated plants. Other authors reported that the total amount of organic acids decreases under osmotic stress in bean leaves (Sassi et al., 2010). On the other hand, drought does not trigger the accumulation of organic acids except for succinate in soybean (Silvente, Sobolev & Lara, 2012). We observed a lower level of citric acid in our plants treated with calcium. Some researchers report that citric acid can improve photosynthetic rates, reduce reactive oxygen species, and provide better osmoregulation under drought stress (Tahjib-Ul-Arif et al., 2021). Therefore, additional studies are necessary to evaluate the effect of drought stress on the organic acid fluctuations in tree crops.

### The influence of Ca application on gene expression profiles of the tea plant under drought and relations with biochemical data

Most of crop quality–related genes proved to be downregulated under drought stress in the tea plant, in line with other studies (Li et al., 2015; Li et al., 2019; Wang et al., 2016a). On the contrary, several stress-related genes were upregulated by drought in both control and Ca-treated plants, in agreement with other research pointing to their involvement in stress responses (Wang et al., 2016b; Liu et al., 2016b; Samarina et al., 2020; Wrzaczek et al., 2010; Tanaka et al., 2012; Paul & Kumar, 2013; Li et al., 2016; Ban et al., 2017; Cheng et al., 2016; Yin et al., 2022).

Among the upregulated genes, three (*LOX1*, *RHL41*, and *GOLS1*) showed 2–3 times greater relative expression in Ca-treated plants as compared to control plants. GolS is the key enzyme for the synthesis of raffinose family oligosaccharides, which serve as osmoprotectants in plant cells and protect salicylate from an attack by hydroxyl radicals (*e.g*., galactinol and raffinose perform this function) (Panikulangara et al., 2004; Nishizawa, Yabuta & Shigeoka, 2008; Falavigna et al., 2018; Li et al., 2019). The plants overexpressing *GOLS1* accumulate galactosyl inositol, which acts as a sugar signal in the ethylene signaling cascade (Li et al., 2019). *GolS1*- or *GolS2*-overexpressing *Arabidopsis thaliana* has high intracellular levels of galactinol and raffinose, which correlate with higher tolerance of drought stress (Panikulangara et al., 2004). Based on our results, it can be hypothesized that calcium affects *GOLS1* expression and participates in the galactinol biosynthesis pathway leading to better acclimation of the tea plant to drought.

*RHL41* (responsive to high light) is a member of the *C2H2* family and is related to zinc-finger protein Zat12. Transgenic *Arabidopsis* plants overexpressing *RHL41* possess thick dark green leaves and higher anthocyanin and chlorophyll contents (Iida et al., 2000). *RHL41* plays a critical part in salt and drought responses by participating in the ABA-dependent pathway (Miller, Shulaev & Mittler, 2008; Ghorbani, Alemzadeh & Razi, 2019; Samarina et al., 2020). In our previous study, increased accumulation of *RHL41* transcripts was observed, indicating specific involvement of this gene in drought defense (Samarina et al., 2020). In the present study, Ca treatment enhanced the upregulation of *RHL41* in the tea plant, pointing to a stronger ABA-mediated response to drought; this phenomenon can explain better protection of membranes from oxidative stress.

The *LOX* gene family is known to be involved in lipid catabolism for oxylipin synthesis playing an important role in the JA-dependent pathway in various stress responses (Liavonchanka & Feussner, 2006). *CsLOX1* is induced by a pathogen infection and brief cold treatment in the tea plant and partakes in the JA-responsive pathway (Zhu et al., 2018). In our work, calcium treatment affected the upregulation of *LOX1*, meaning the activation of the ABA-independent stress response. This finding is consistent with data from Montillet et al. (2013), who demonstrated that *LOX1* performs a major function in the control of stomatal defense and plant innate immunity. They reported that the activities of an oxylipin- and an ABA-dependent pathway converge on anion channel SLAC1 thereby regulating stomatal closure. Thus, we can speculate that calcium participates in the regulation of stomatal aperture in guard cells not only through activation of ABA signaling but also through oxylipin signaling activation by inducing the *LOX1* expression. This theory is supported by PCA: the positive association of Met and Val contents with the *LOX1* gene and high positive loading along the Ca vector observed in PCA (Fig. 4) confirmed the important role of calcium in the JA-mediated drought response in the tea plant. As mentioned above, under drought stress, Met and Val accumulated more in Ca-treated tea plants, and these amino acids participate in JA pathway activation.

Notably, in our study, three stress-related transcription factors (*bHLH102*, *WRKY2*, and *WRKY42*) were downregulated by drought stress in both control and Ca-treated plants. These genes are involved in stress and hormone signaling, particularly in the ABA-mediated abiotic-stress response (Wang et al., 2016a; Phukan, Jeena & Shukla, 2016; Jiang et al., 2017; Cui et al., 2018). This contradictory result may be due to genotype-specific responses or dissimilar stress conditions used in various studies. A limitation of our study is that we did not evaluate a short-term drought response. Further investigation will help to assess temporal and spatial expression alterations of the aforementioned genes.

## Conclusions

Effects of external Ca application on drought responses of the tea tree were evaluated. Under drought, a greater increase in cell sap pH; higher accumulation of Tyr, Met, and Val; greater contents of the malic and citric acids; and higher Mg^2+^ concentration were observed in Ca-treated tea plants. Among the upregulated genes, three genes (*LOX1*, *RHL41*, and *GOLS1*) showed 2–3 times greater relative expression in Ca-treated plants than in control plants. PCA results indicate a positive correlation of Met and Val contents with *LOX1* mRNA expression, confirming the important function of calcium in the activation of JA signaling in the tea plant under drought stress. On the basis of these results, it can be theorized that calcium affects the galactinol biosynthesis pathway and participates in the regulation of stomatal aperture in guard cells not only through ABA signaling activation but also through oxylipin pathway activation. Thus, calcium improves the drought response in the tea tree. These findings improve our understanding of calcium-mediated drought defense in tree crops. Further studies will reveal temporal and spatial changes of expression of the above-mentioned genes.

## Supplemental Information

10.7717/peerj.13997/supp-1 Supplemental Information 1 Tea plant genes and primers.

10.7717/peerj.13997/supp-2 Supplemental Information 2 Three-year-old vegetatively propagated potted plants of the best local tea cultivar Kolkhida were used in this study. Plants were 50 cm tall and grown in 2-liter polyethylene pots filled with brown forest acidic soil.

10.7717/peerj.13997/supp-3 Supplemental Information 3 Raw data. Experimental measurements for the treated and control plants (pH, water, sugar, amino acid and cation content shown in Figures 1 and 2).

## Additional Information and Declarations

### Data Availability

The following information was supplied regarding data availability: The raw data is available in the Supplemental Table.
