# Supplementary figures and images for: Effects of exogenous calcium on the drought response of the tea plant (Camellia sinensis (L.) Kuntze)
Source: PeerJ. 2022 Aug 29;10:e13997. doi: 10.7717/peerj.13997 (PMC9435517; doi:10.7717/peerj.13997)

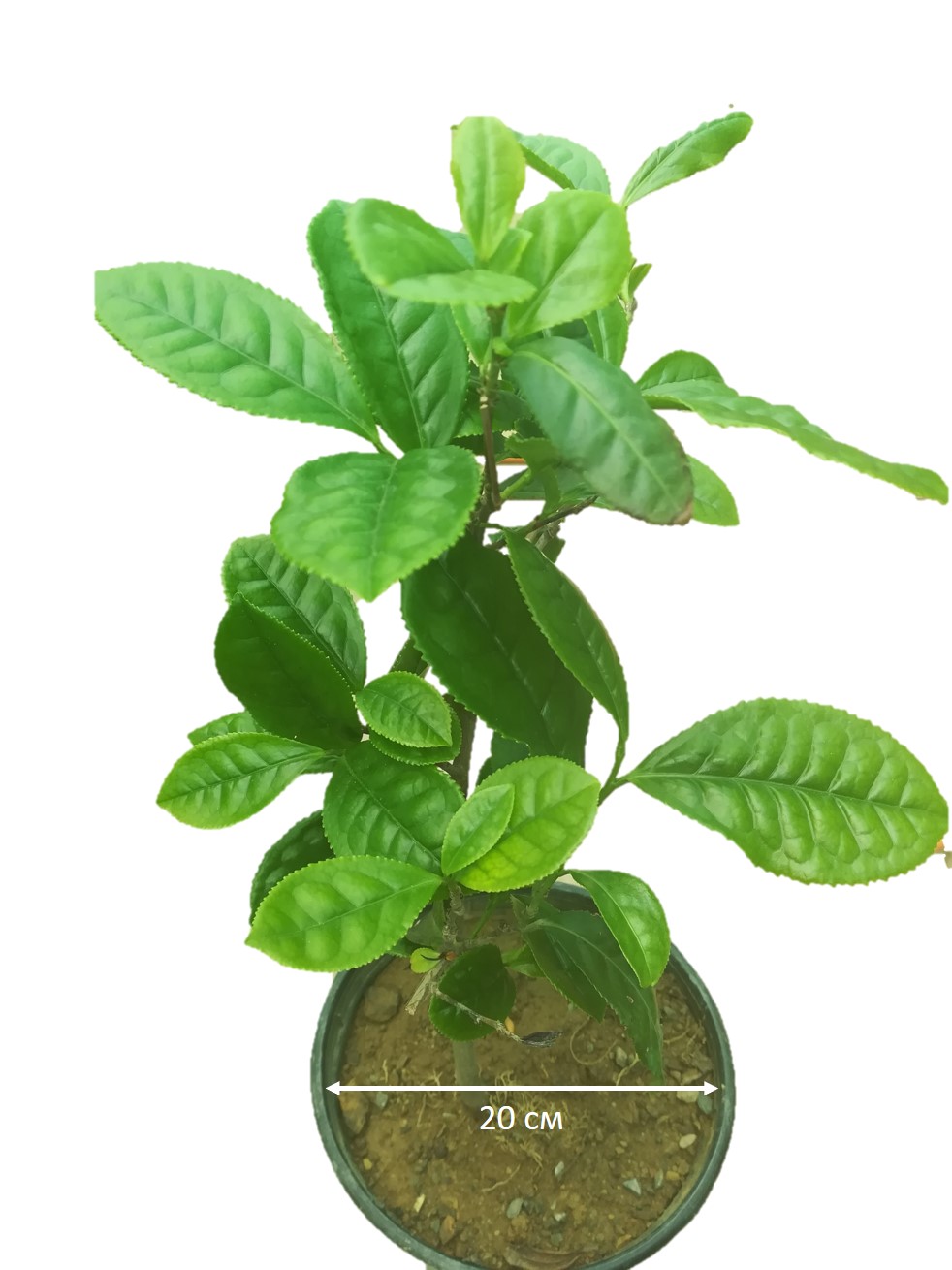

Supplement: Supplemental Information 2 — Plants were 50 cm tall and grown in 2-liter polyethylene pots filled with brown forest acidic soil. [file peerj-10-13997-s002.jpg]
